# Curation of microarray oligonucleotides and corresponding ESTs/cDNAs used for gene expression analysis in zebra finches

**DOI:** 10.1186/s13104-018-3402-x

**Published:** 2018-05-18

**Authors:** Peter V. Lovell, Nicole A. Huizinga, Abel Getachew, Brianna Mees, Samantha R. Friedrich, Morgan Wirthlin, Claudio V. Mello

**Affiliations:** 1 Department of Behavioral Neuroscience, OHSU, Portland, OR 97221 USA; 2 Present Address: Computational Biology, Carnegie Mellon University, Pittsburgh, PA USA

**Keywords:** Molecular, Speech and language, Birdsong, cDNA microarray, Oligo array, Gene expression, Brain, Vocal learning

## Abstract

**Objectives:**

Zebra finches are a major model organism for investigating mechanisms of vocal learning, a trait that enables spoken language in humans. The development of cDNA collections with expressed sequence tags (ESTs) and microarrays has allowed for extensive molecular characterizations of circuitry underlying vocal learning and production. However, poor database curation can lead to errors in transcriptome and bioinformatics analyses, limiting the impact of these resources. Here we used genomic alignments and synteny analysis for orthology verification to curate and reannotate ~ 35% of the oligonucleotides and corresponding ESTs/cDNAs that make-up Agilent microarrays for gene expression analysis in finches.

**Data description:**

We found that: (1) 5475 out of 43,084 oligos (a) failed to align to the zebra finch genome, (b) aligned to multiple loci, or (c) aligned to Chr_un only, and thus need to be flagged until a better genome assembly is available, or (d) reflect cloning artifacts; (2) Out of 9635 valid oligos examined further, 3120 were incorrectly named, including 1533 with no known orthologs; and (3) 2635 oligos required name update. The resulting curated dataset provides a reference for correcting gene identification errors in previous finch microarrays studies, and avoiding such errors in future studies.

## Objective

Zebra finches represent a major model organism for studying vocal learning [1–6], a trait that provides a basis for spoken language acquisition in humans. Studies in finches have led to insights into the molecular machinery that underlies learned vocalizations [7–19], including the transcriptome of the vocal control circuitry [7, 8, 11–16, 18–25] and the identification of convergent molecular specializations of the vocal control systems of birds and humans [7]. Such studies were largely based on the Songbird array v2 [16], a ~ 44,000 60-mer oligonucleotide array designed with eArray 5.4 (Agilent Technologies) and sequences from three cDNA collections [11, 16, 23]. Initial cDNA annotations were made before the zebra finch genome was available through BLAST searches of annotated cDNA/EST databases. Later efforts aligned oligo and EST sequences to the zebra finch genome (Taegut1; [26]), and assigned Ensembl model annotations to oligos that mapped to within 5 kb (or ESTs within 3 kb) of those models [7, 25]. However, this effort did not take into account strand information, did not detect ESTs/oligos intronic to gene models, or assigned ESTs/oligos to models that were incorrectly annotated. Other oligos were derived from cDNA cloning artifacts, or erroneous sequence selection. By removing and correcting these errors, we generated what we consider the most thorough and accurate constitutive transcriptome of the zebra finch song control system [27]. We describe this curation effort below.

## Data description

We retrieved the full set of oligos (60-mers) from the Agilent-021323 Zebra Finch Oligoarray (https://www.ncbi.nlm.nih.gov/geo/query/acc.cgi?acc=GPL18442), removing redundancies and controls. For 43,084 non-redundant oligos we applied a similar curation effort as described in [9, 28, 29]. Table 1 provides links to a summary of our curation effort (Table 2), and relevant datasets (Tables 3–13). The complete collection of datasets can be found at 10.6084/m9.figshare.c.4081835 [30]. We first aligned all oligos to the finch genome (Taegut1) using BLAT [31] with stringent parameters (minScore = 30; minIdentity = 0). 2792 oligos (6%) failed to align to Taegut1 (alignment score < 25; Table 3), 503 (1%) only aligned to Chr_Un (i.e., chromosome unknown; Table 4), a concatenation of unassembled regions and allelic variants, and 1952 (5%) aligned to multiple loci on different chromosomes (Table 5). All cases above were removed from further analyses, as one cannot determine specificity or establish gene orthology based on synteny. We retained oligos with high scoring (> 95%) secondary alignments to Chr_Un, since these correspond to allelic variants. Another 228 oligos (< 1%) were in opposite orientation to ESTs sequenced from the 5′ end of the cDNAs, or in the same orientation as ESTs sequenced from the 3′ end (Table 6). These were also removed as they represent antisense strands of short ESTs with T-stretches at both ends due to second-strand oligo-dT priming and non-directional cDNA cloning.

**Table 1:** Overview of data files/data sets

Label	Name of data file/data set	File types (file extension)	Data repository and identifier (DOI or accession number)
Data file 1	Table 2	MS Excel file (.xlsx)	10.6084/m9.figshare.6189485
Data file 1	Table 3	MS Excel file (.xlsx)	10.6084/m9.figshare.6189482
Data file 1	Table 4	MS Excel file (.xlsx)	10.6084/m9.figshare.6189479
Data file 1	Table 5	MS Excel file (.xlsx)	10.6084/m9.figshare.6189476
Data file 1	Table 6	MS Excel file (.xlsx)	10.6084/m9.figshare.6189470
Data file 1	Table 7	MS Excel file (.xlsx)	10.6084/m9.figshare.6189467
Data file 1	Table 8	MS Excel file (.xlsx)	10.6084/m9.figshare.6189461
Data file 1	Table 9	MS Excel file (.xlsx)	10.6084/m9.figshare.6189452
Data file 1	Table 10	MS Excel file (.xlsx)	10.6084/m9.figshare.6189446
Data file 1	Table 11	MS Excel file (.xlsx)	10.6084/m9.figshare.6189440
Data file 1	Table 12	MS Excel file (.xlsx)	10.6084/m9.figshare.6189437
Data file 1	Table 13	MS Excel file (.xlsx)	10.6084/m9.figshare.6189431

For 27,974 out of the 37,609 oligos (74%) that passed initial filters we provide the consensus gene symbol as in previous efforts [7, 32] (Table 7), based on the Human Genome Nomenclature Consortium (HGNC; 2018). For the remaining 9635 oligos (26%) that define the constitutive transcriptome of the finch song system [27], we inspected alignments against Taegut1, and annotated sequences based on association with a gene model (Ensembl or finch-/xeno-RefSeqs on the correct strand). For ESTs corresponding to 3′-UTRs, we BLAT-aligned sequences to chicken (*Galgal5*) to try to connect them to chicken gene models by ‘walking’ the extensive chicken ESTs/mRNAs collection. In total, 3750 oligos annotations were confirmed by direct inspection (Table 8), and an additional 130 oligos further confirmed by synteny (Table 9), which required additional alignments and neighbor gene comparisons with other avian (e.g. chicken, Tibetan tit, other finches, budgerigar, starling, falcon) and non-avian (i.e., alligator, lizard, mouse, human) genomes. We provided correct annotations for 1529 unannotated or misannotated oligos (Table 10), including cases of improper Ensembl model assignment (e.g. wrong strand) or intronic location to a model, determining orthology for another 58 oligos (Table 11). 1533 oligos associated with loci with no orthologs in other organisms (Table 12) were named unknown. Lastly, we updated 2635 oligos to an HGNC symbol, or a consensus NCBI:Gene name (Table 13).

Our findings highlight the need for accurate curations to avoid propagating errors in gene identification and bioinformatics. This partial curated dataset (~ 35% of oligos on this array) serves as a reference for correcting errors from previous studies, and a roadmap for future oligo curations. We anticipate for the 27,975 oligos not examined here, 32% will require further curation, and 27% will require updated gene symbols (Table 7).

## Limitations

- In our experience, accurate orthology assignment requires synteny verification, however there are no adequate computational methods for large scale analyses, and manual assessment of a large gene set is beyond a reasonable scope of effort. We recommend that caution should be exerted and direct synteny verification be applied whenever deciding to focus on one or a few genes from microarray screenings. This is particularly important in cases of suspected paralogy or sequence cross-alignments to close family members.
- The HGNC annotation step is important since most bioinformatics pipelines use these approved symbols. Here we downloaded the entire set of HGNC gene symbols along with any older gene symbols or synonyms and cross-referenced the lists to verify that the gene symbols of our curated oligo sets were approved terms by HGNC. In most cases, we were able to update older gene symbols or synonyms to a current HGNC gene symbol. In some cases, however, particularly when the zebra finch gene does not have a human ortholog, there is no approved HGNC gene symbol. In these cases, we consulted NCBI:Gene and assigned the gene symbol most commonly shared amongst multiple non-human vertebrates (e.g. mouse, anole lizard, chicken, frog). These NCBI:Gene names are listed as ‘Not Approved’ under the column heading “HGNC Symbol Status” in Tables 7–13 and are not valid entries for bioinformatics applications based on approved human gene terms.

## Abbreviations

BLAT: BLAST-like alignment tool
BLAST: Basic Local Alignment Search Tool
cDNA: DNA synthesized from a single stranded mRNA
chr_Un: chromosome unknown
EST: expressed sequence tags
HGNC: Human Genome Nomenclature Consortium
NCBI: National Center for Biotechnology Information
Xeno-/Refseqs: annotated and curated nucleotide sequences (DNA, RNA)
